# The Value and Risks of Water Exercise

**Published:** 1914-04-04

**Authors:** 


### SWIMMING IN SCHOOL THERAPY.

### The Value and Risks of Water Exercise.

Every school doctor, in the course of his routine work, will have occasion to interest himself in the swimming classes of certain of his schools. Such interest is, for the most part, direct, resulting from the necessity to answer the teacher's inquiries whether or not particular children are fit to participate in the work of the class. In most schools it is the rule that every pupil above the age of eleven shall take part in swimming lessons. This merely means that every pupil is made to accompany the class to the baths and to enter the water; it does not necessarily mean that he or she is systematically taught swimming. Where the class-master is enthusiastic and takes a keen interest in the matter, the percentage of pupils who, in the course of a term, learn to swim, is fairly high; where the master is apathetic, the percentage is necessarily low. It is somewhat difficult to understand the reason for the rule that children below the age of eleven should not attend these classes. In London, we believe, the reason is that the accommodation of the baths is limited, and these junior children are regarded as a nuisance by the older ones. Nevertheless, it is reasonable to suppose that in special cases an exception might be made in favour of younger children. In practice, however, such an exception must be extremely rare, for the Education Committee of the London County Council firmly adhere to their rule that children below the required age shall not be permitted to learn to swim under the Council's auspices. From a purely medical point of view such a rule is nonsense. As Professor Spitzy has shown, the young child learns swimming much more easily than the older child; as soon as a child is able to walk he should also be taught to swim; the one method of progression is as natural as the other, and there is no reason whatever why both should not be taught in infancy.

The children who are not fit to attend the ordinary swimming classes fall into two main categories; children whose own welfare would be risked by allowing them to swim, and children whose presence in the swimming bath would entail risk to other swimmers. Sometimes a child falls under both categories, as in the case of a child with discharging ears, but usually the prohibition against joining the class is based on the assumption that it is not in the pupil's interests that he or she shall swim. These "risky" cases, belonging to the first group, are epileptic children, those with grave cardiac disease, with tuberculosis of the lungs or bowels, and with nervous signs. To these one might as reasonably add children whose blood pressure is high, who have aural or thoracic disease, who suffer from recurrent attacks of epistaxis or from nystagmus. As a matter of fact none of these children need be absolutely debarred from swimming; all that is necessary in their case is that they should be carefully supervised, that the master or mistress in charge of the class should know exactly what dangers are to be feared, and how they are to be guarded against, and that the child should not be allowed to suffer through over-exertion. With these precautions children suffering from any of the defects stated may be permitted to swim, with the possible exception of really bad heart disease and epileptic children.

Heart disease is usually regarded as an absolute bar to participation in the swimming exercises. Where, however, there is well-established compensation, swimming, in moderation, is no more objectionable for these children than is walking or running; moderation and care must be the rule rather than absolute prohibition. It is unjustifiable to exclude a child from the swimming classes merely because he or she has a systolic apical murmur or slight mitral disease. Aortic disease—which is, however, very rare in children—is another matter; here the care should be extreme, and swimming is not justified; such a child, however, should not be in an ordinary school but should have special attention paid to it. As a rule it may be taken that where a child is debarred from swimming in its own interests that child should not be allowed to attend an ordinary school, since his or her case is sufficiently serious to warrant extra care and attention.

In the second group are included children suffering from any contagious disease or from any affection which is likely to pollute the water. Such are children afflicted with skin diseases—psoriasis, eczema, impetigo, scabies, seborrhœic dermatitis, or ringworm; children with bad rhinitis, otorrhœa, vaginitis, ciliary blepharitis, ulceration, discharging sinuses, or incontinence; and, of course, all infectious diseases. Sufficient care is not always taken to prevent such children from contaminating the baths; in fact, it is by no means unusual to see children excluded from school by reason of their being afflicted with one of these diseases, occupying some of their enforced leisure time by larking in the baths. Bath attendants should have definite instructions not to admit children of school-going age who are unaccompanied by instructors or masters without some certificate of freedom from infection or offensiveness; this is, no doubt, a counsel of perfection, but some such rule is urgently needed. Recent investigations of the water of certain swimming baths have shown that they are by no means bacteriologically pure, and, although it is not always easy to prove that some disease has been contracted at the bath, there is enough evidence to show that such infection is extremely probable in a large number of cases.

Indirectly the school doctor should interest himself in the matter of swimming, because this exercise is a very useful adjuvant in the treatment of certain conditions. Of these the most important is undoubtedly lateral curvature. This is very frequent in school children, especially in a minor degree where the curvature, a postural or fatigue curvature, is so slight as to make the parents disregard the necessity for treatment. To send such a child to a hospital is to court disaster; the parents are usually dismissed with a curt intimation that "there is nothing serious the matter with the child"; at best, in the orthopædic hospitals where incipient lateral curvature is recognised and treated, they are told to bring the child so many times a week for treatment in the Zander room or for massage. Few parents are willing to undertake to do this in cases where they do not grasp the importance of dealing with these early cases of scoliosis; again, the child, by such attendance, misses the school marks which it so much prizes. In the circumstances the school doctor should recommend swimming exercises, especially the breast stroke; this, regularly pursued, under the supervision of an intelligent master or mistress who has been made aware of the child's defect, generally leads to rapid improvement, while it entails no loss of attendance marks for the child. Swimming is also highly useful in cases of weakly, undersized children, especially of the class styled pretubercular; the exercise, and perhaps also the therapeutic effect of the bath, act marvellously in many cases. This is no doubt largely the result of exercising a different set of muscles, of the deep breathing, and consequent extra aeration of the blood, induced by swimming, and probably also of the stimulating effect of the water on the skin.

